# Supplementary material for: Bioengineered bacteria-derived outer membrane vesicles as a versatile antigen display platform for tumor vaccination via Plug-and-Display technology
Source: Nat Commun. 2021 Apr 6;12:2041. doi: 10.1038/s41467-021-22308-8 (PMC8024398; doi:10.1038/s41467-021-22308-8)
Supplement: Supplementary file 1 — Supplementary Information [file 41467_2021_22308_MOESM1_ESM.pdf]

Supplementary Information

Bioengineered bacteria-derived outer membrane vesicles as a versatile antigen display platform for tumor vaccination *via* Plug-and-Display technology

Keman Cheng[1,2,3#], Ruifang Zhao[1,2#], Yao Li[1,2,3], Yingqiu Qi[1,2], Yazhou Wang[1,2], Yinlong Zhang[1,2], Hao Qin[1,2], Yuting Qin[1,2], Long Chen[1,2], Chen Li[1,2], Jie Liang[1,2], Yujing Li[1,2], Jiaqi Xu[1,2], Xuexiang Han[1,2], Gregory J. Anderson[4], Jian Shi[1,2], Lei Ren[3], Xiao Zhao[1,2*], Guangjun Nie[1,2*]

[1] CAS Key Laboratory for Biomedical Effects of Nanomaterials and Nanosafety & CAS Center for Excellence in Nanoscience, National Center for Nanoscience and Technology of China, 11 Beiyitiao, Zhongguancun, Beijing, 100190, China

[2] Center of Materials Science and Optoelectronics Engineering, University of Chinese Academy of Sciences, Beijing 100049, China

[3] Department of Biomaterials, Key Laboratory of Biomedical Engineering of Fujian Province, College of Materials, Xiamen University, Xiamen, Fujian 361005, China

[4] Iron Metabolism Laboratory, QIMR Berghofer Medical Research Institute, Brisbane, Queensland 4006, Australia

[*] Corresponding authors:
Xiao Zhao: zhaox@nanoctr.cn, Tel: +86-10-82545529, Fax: +86-10-62656765
Guangjun Nie: niegj@nanoctr.cn, Tel: +86-10-82545529, Fax: +86-10-62656765

[#] The authors contributed equally to this paper.

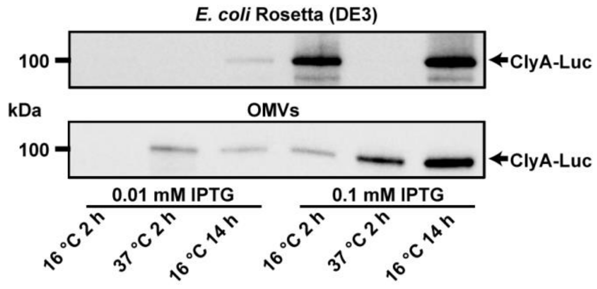

Supplementary Figure 1. Optimization of the conditions for expressing ClyA-Luc (CL) in OMVs. As protein expression in bacteria is affected mainly by culture temperature, culture time, and the concentration of the inducer (IPTG), we investigated expression at two temperatures, over two time periods, and at two inducer concentrations to determine the best expression conditions. Western blot analysis was used to assess the expression of CL. Source data are provided as a Source Data file.

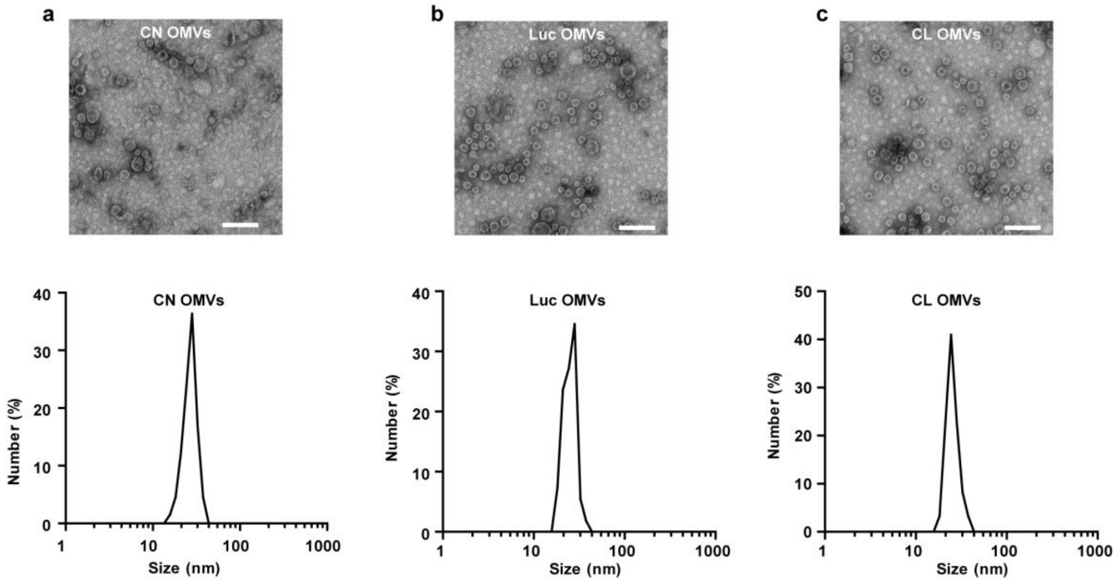

Supplementary Figure 2. Characterization of morphology and particle size of OMVs. TEM images and DLS analysis of (a) ClyA-none (CN) OMVs, (b) Luc OMVs and (c) ClyA-Luc (CL) OMVs. Scale bar, 100 nm. Source data are provided as a Source Data file.

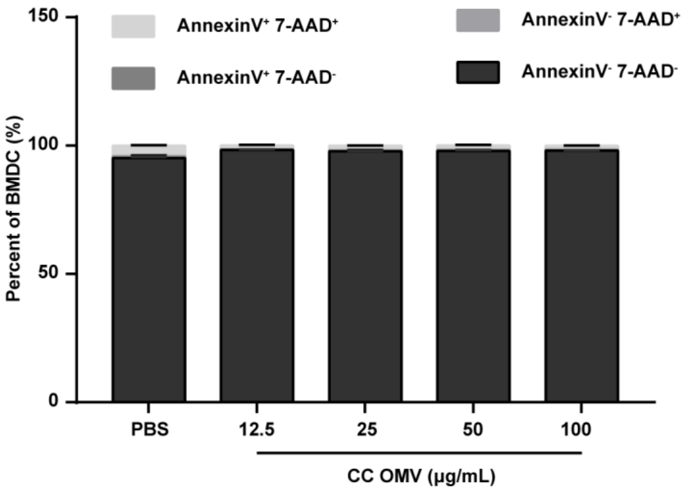

Supplementary Figure 3. Cytotoxicity evaluation of OMVs. The cytotoxicity of OMVs in murine bone marrow-derived dendritic cells (BMDCs) was measured by flow cytometry after 24 h incubation with CO OMVs at the indicated protein concentrations or PBS. Cells were stained with annexin V-APC/7-AAD. The Annexin V^-/7-AAD$^-$ cells are viable cell. The data were shown as mean ± SD (n = 4). Source data are provided as a Source Data file.

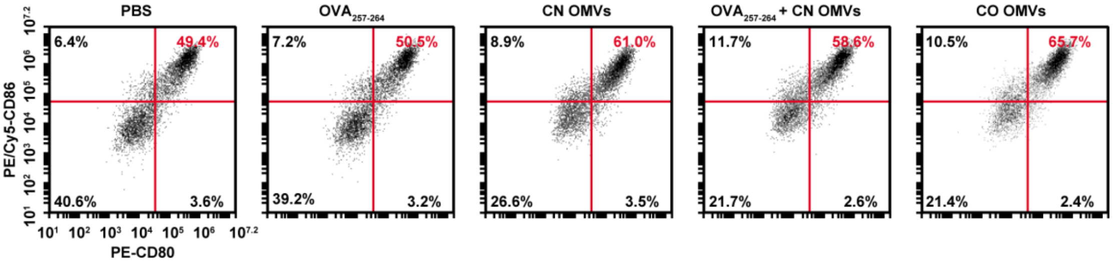

Supplementary Figure 4. Evaluation of DC maturation stimulated by OMVs (related to Figure 2a-b). Representative flow dot plots of $CD80^+$ or $CD86^+$ cells in $CD11c^+$-gated BMDCs, as analyzed by flow cytometry.

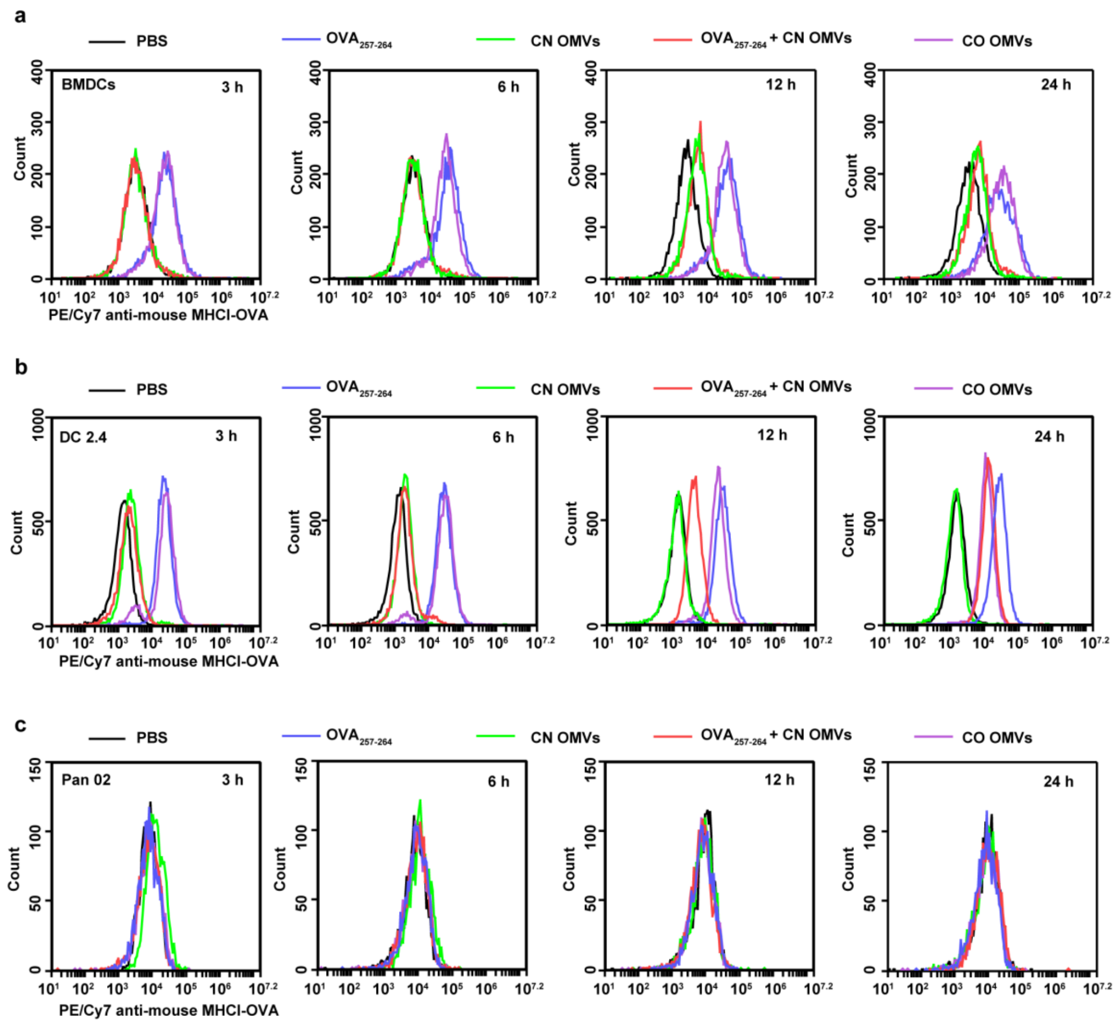

Supplementary Figure 5. Evaluation of antigen presentation in DCs and Pan 02. Representative histograms of MHCI-OVA^{+} (MHCI H-2Kb bound to $OVA_{257\text{-}264}$) BMDCs (a), DC 2.4 cells (b) and Pan 02 cells (c) treated with the indicated formulations for the indicated time intervals. The antigen was only presented by the DCs (BMDCs and DC2.4).

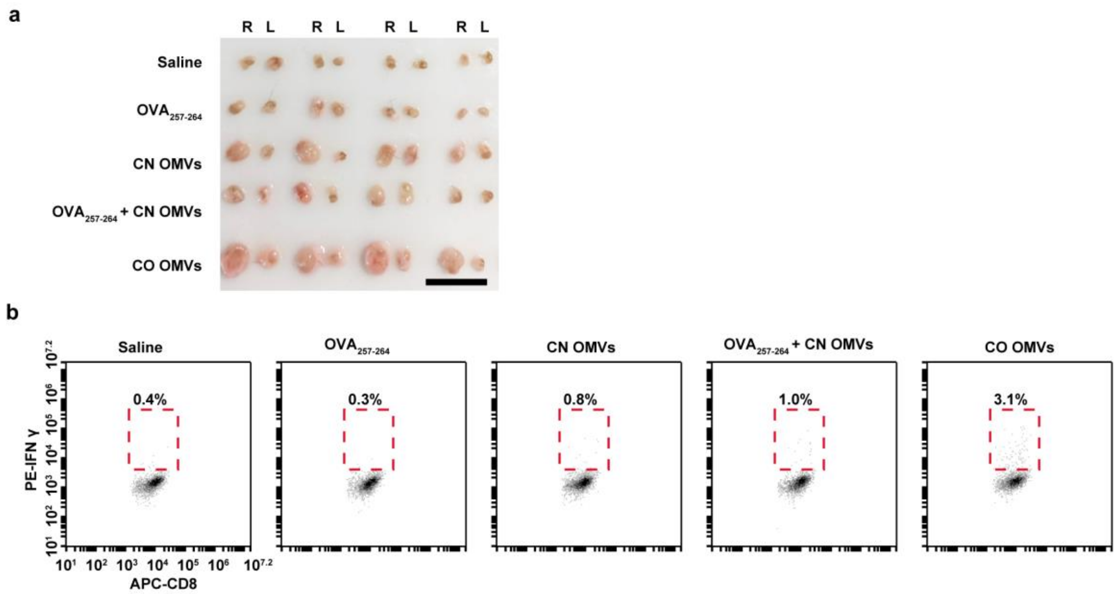

Supplementary Figure 6. Antigen-specific immune response induced by CO OMVs (related to Figure 2g and Figure 2l). (a) The lymph nodes of mice (n = 4) on day 17, after challenge with B16-OVA cells and immunization with the indicated formulations, were collected and photographed. Mice received immunization subcutaneously on the right side of the back. Bilateral inguinal lymph nodes were collected. L, left; R, right. Scale bar, 1 cm. (b) Representative flow dot plots of IFNγ^+ cells in CD3$^+$CD8$^+$ T lymphocytes in the spleens of mice on day 17 after immunization with the indictaed OMVs formulations and controls, as analyzed by flow cytometry. Source data are provided as a Source Data file.

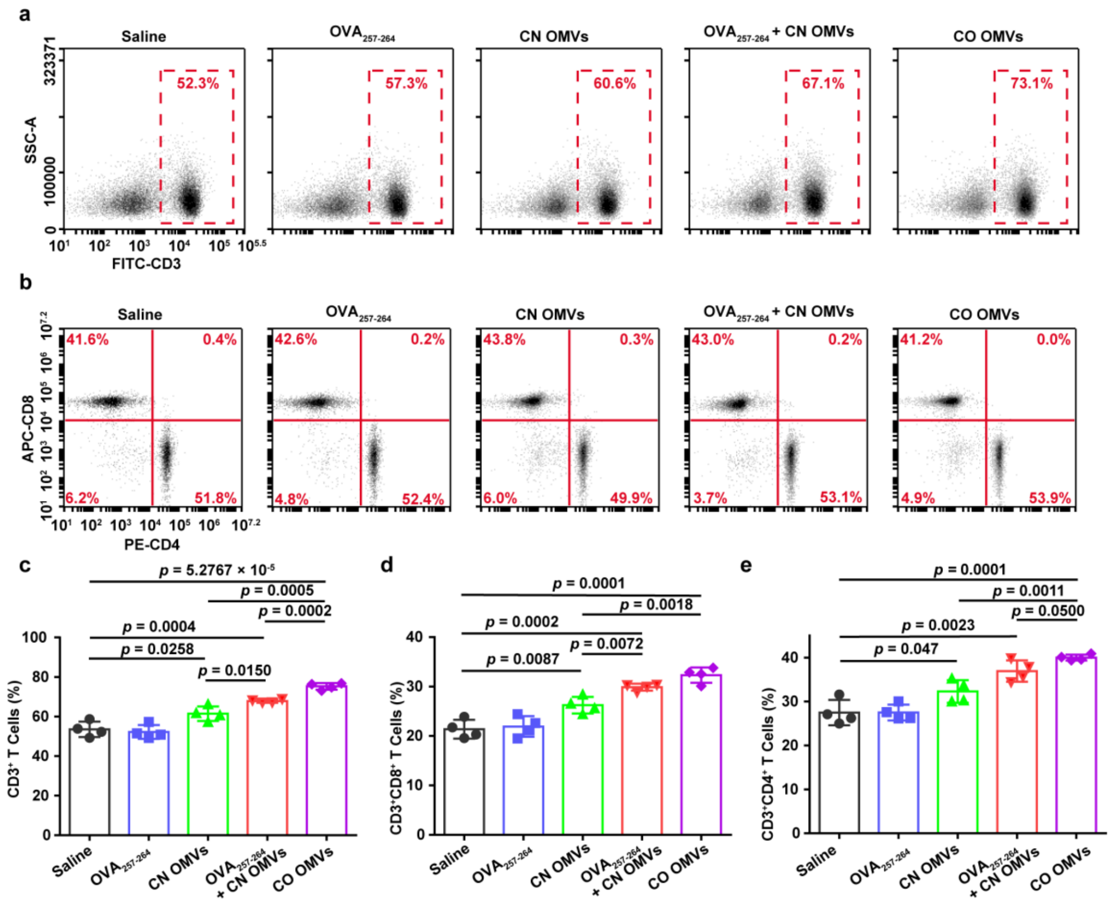

Supplementary Figure 7. Flow cytometry analysis of T cells in draining lymph node (related to Figure 2g). Flow cytometry analysis of T cell populations from the right inguinal lymph nodes of mice on day 17 after challenge with B16-OVA cells and immunization with the indicated formulations. The proportion of $CD3^+$ T lymphocytes is shown in (a) and (c). The proportions of $CD3^+CD8^+$ and $CD3^+CD4^+$ T lymphocytes are shown in (b) and (d), and (b) and (e), respectively. Data represent the mean ± SD (n = 4). Two-tailed unpaired t-test. Source data are provided as a Source Data file.

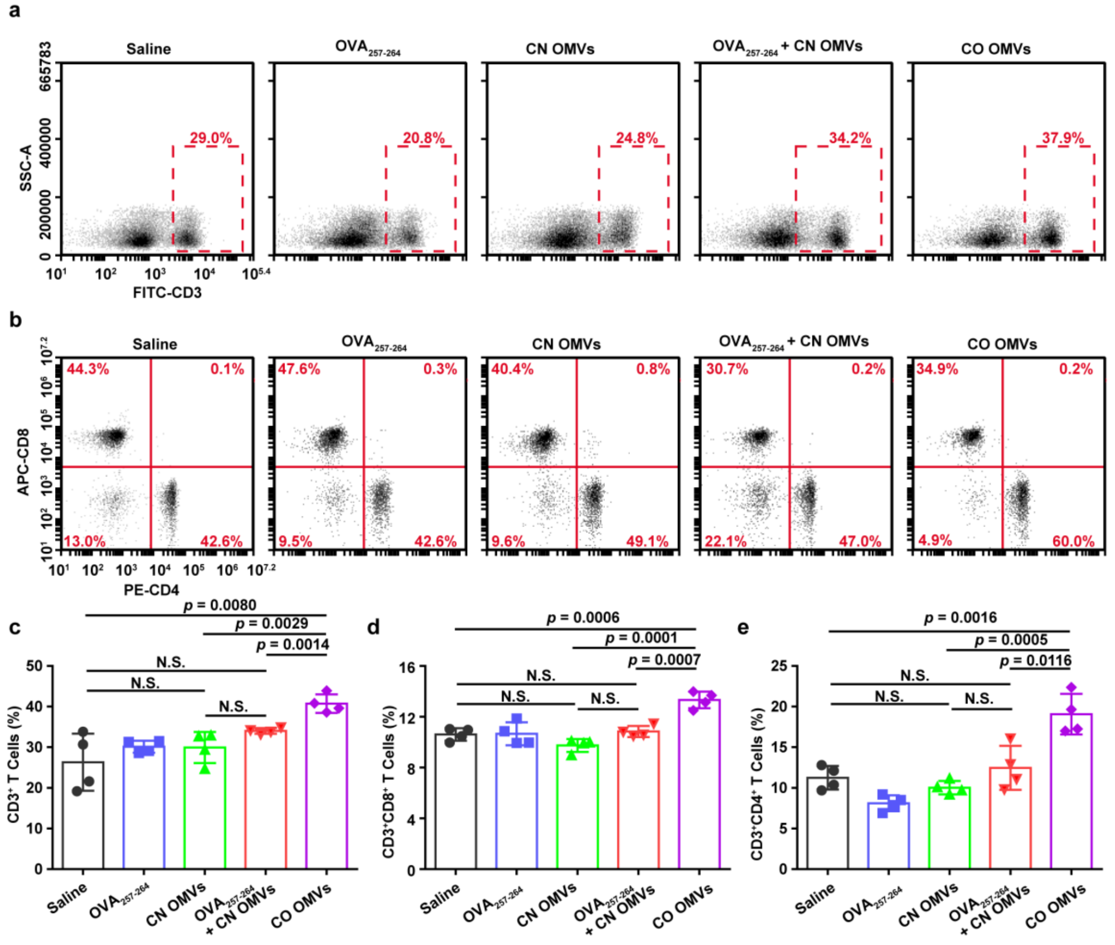

Supplementary Figure 8. Flow cytometry analysis of T cells in blood (related to Figure 2g). Flow cytometry of T cell populations in the blood of mice at day 17 after challenge with B16-OVA cells and immunization with the indicated formulations. The percentage of $CD3^+$ T lymphocytes (a) was determined by flow cytometry, and the percentage of $CD4^+$ or $CD8^+$ cells in $CD3^+$ T lymphocytes was also detected by flow cytometry (b). The percentage of $CD3^+$, $CD3^+CD8^+$, and $CD3^+CD4^+$ T lymphocytes were recorded and plotted in (c), (d) and (e), respectively. Data represent the mean ± SD (n = 4). Two-tailed unpaired t-test. N.S., no significance. Source data are provided as a Source Data file.

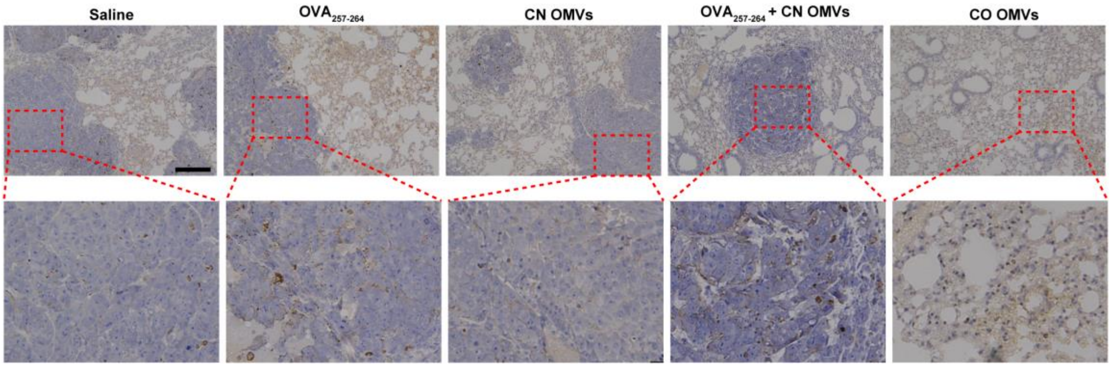

Supplementary Figure 9. The infiltration of CD8$^+$ cells in the lungs (related to Figure 2g). Immunohistochemical staining of CD8$^+$ cells (brown staining) in the lungs of mice at day 17 after challenge with B16-OVA cells and immunization with the indicated formulations. Scale bar, 200 μm.

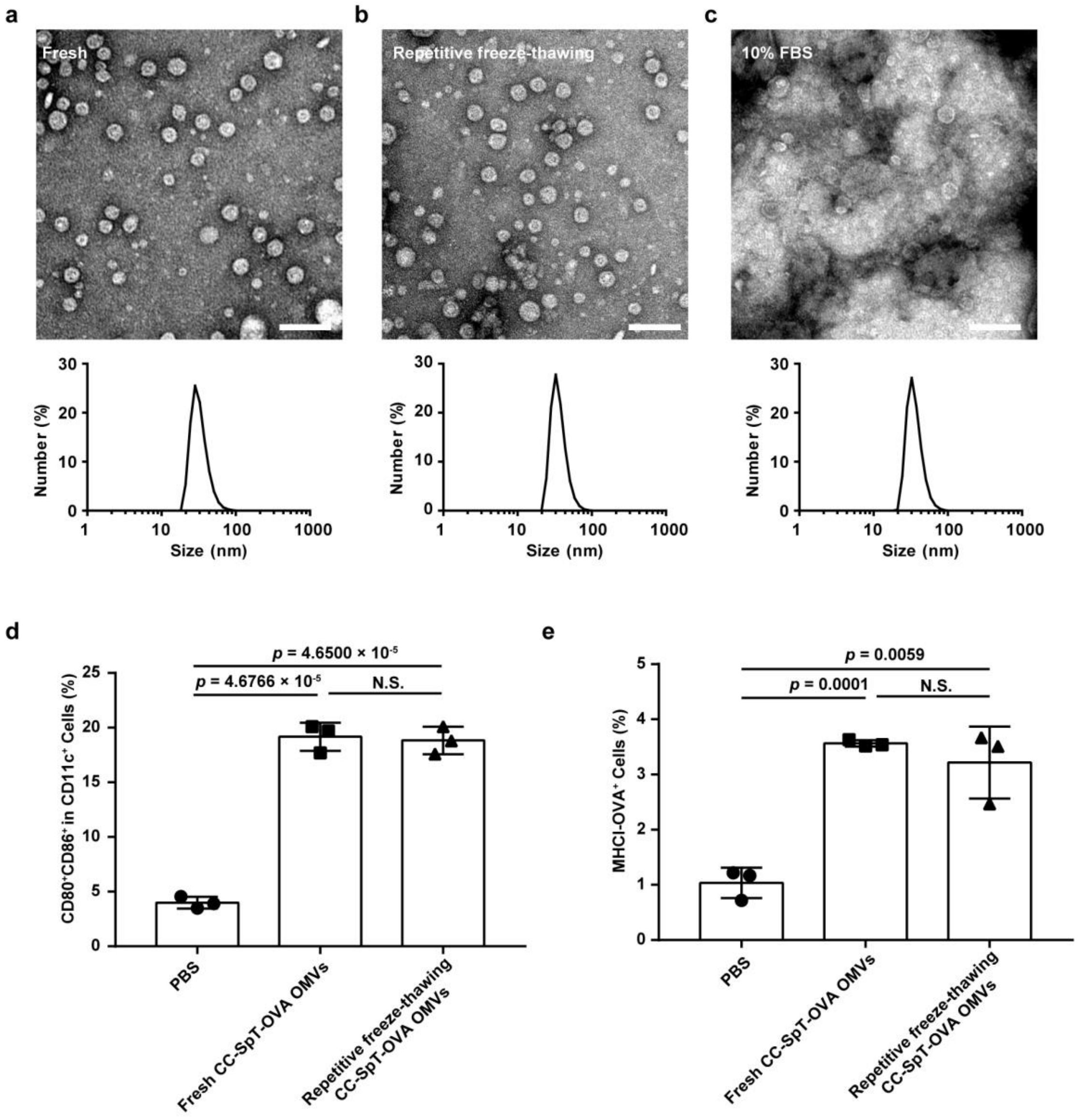

Supplementary Figure 10. The stability of CC OMVs under different conditions. (a)-(c) TEM images and DLS analysis of fresh CC OMVs (a), CC OMVs after 5 freeze-thaw cycles (-80 °C) (b) and CC OMVs after incubation in 10% FBS for 24 h (c). Scale bar, 100 nm. (d) and (e) The analysis of immune stimulation function of different formulation. The expression of the maturation markers $CD80^{+}CD86^{+}$ was examined as a percentage of $CD11c^{+}$ cells by flow cytometry (d). The expression of the MHCI-OVA complex on the surface of BMDCs was measured by flow cytometry ($CD11c^{+}$MHC I-OVA^{+}) (e). All data are presented as mean ± SD (n = 3). Two-tailed unpaired t-test. N.S., no significance. Source data are provided as a Source Data file.

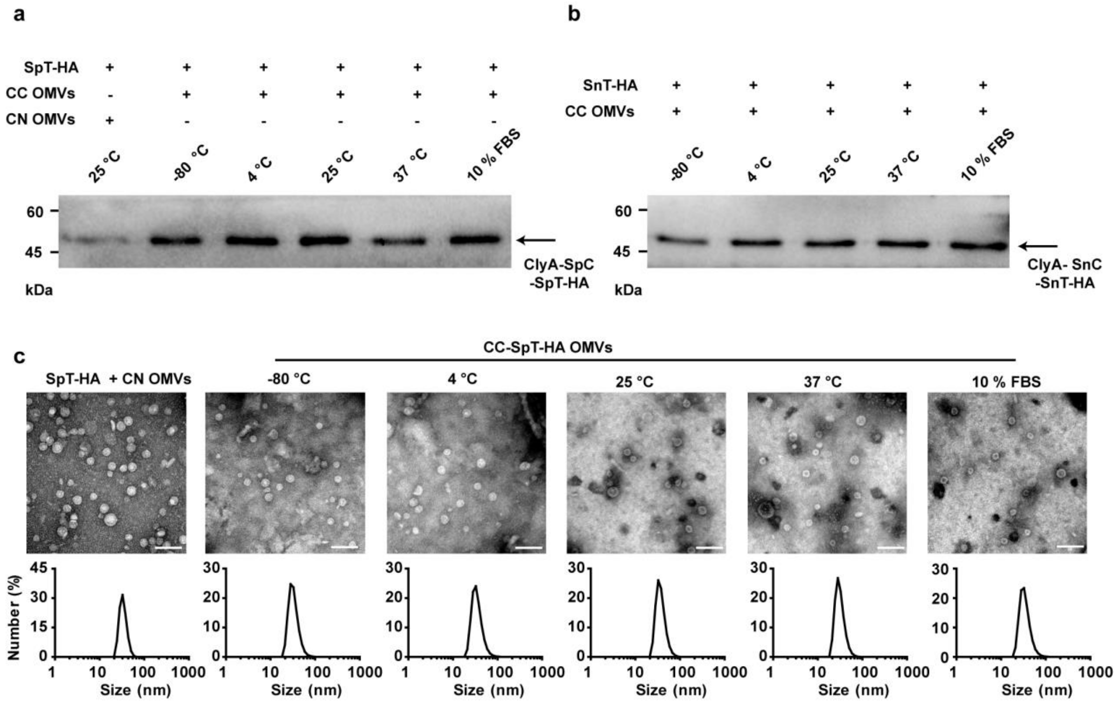

Supplementary Figure 11. The stability of the linkage between SpC and SpT, or SnC and SnT, on the CC OMVs surface under different conditions. (a) and (b) Examination of ClyA-SpC-SpT-HA (a) or ClyA-SnC-SnT-HA (b) complexes by western blot analysis. CC OMVs were incubated with SpT-HA or SnT-HA for 1 h, then stored at different temperatures or treated with 10% FBS for 24 hours. (c) TEM images and DLS analysis of CC-SpT-HA OMVs incubated under different conditions. Scale bar, 100 nm. Source data are provided as a Source Data file.

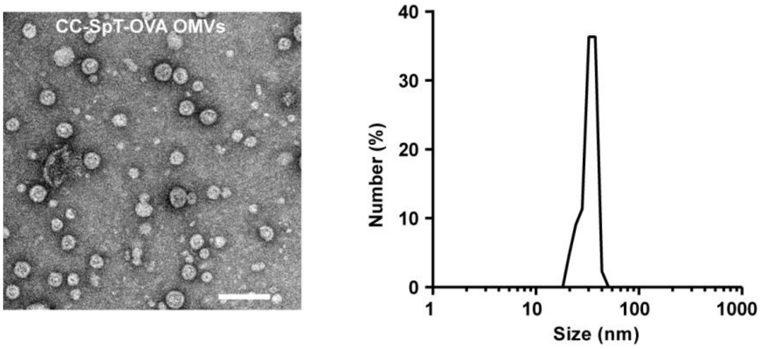

Supplementary Figure 12. TEM image and DLS analysis of CC-SpT-OVA OMVs. Scale bar, 100 nm. Source data are provided as a Source Data file.

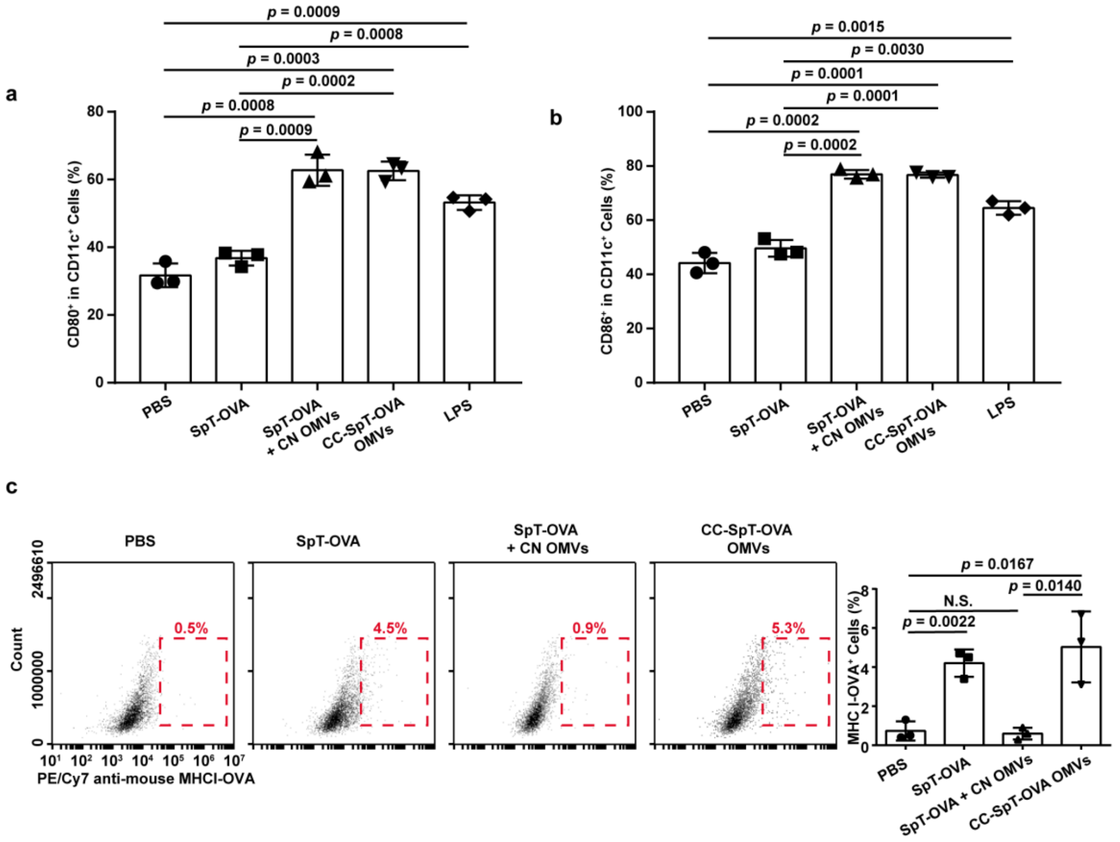

Supplementary Figure 13. DC maturation and antigen presentation induced by CC OMVs. (a) and (b) Maturation of BMDCs after the indicated treatments. The expression of the maturation markers $CD80^+$ (a) or $CD86^+$ (b) was examined as a percentage of $CD11c^+$ cells by flow cytometry. LPS was used as positive control. (c) Representative flow dot plots and corresponding quantitative data of $MHI\text{-}OVA^+$ cells in BMDCs treated with the indicated formulations. All data are presented as mean ± SD (n = 3). Statistical analysis was performed by two-tailed unpaired t-test. N.S., no significance. Source data are provided as a Source Data file.

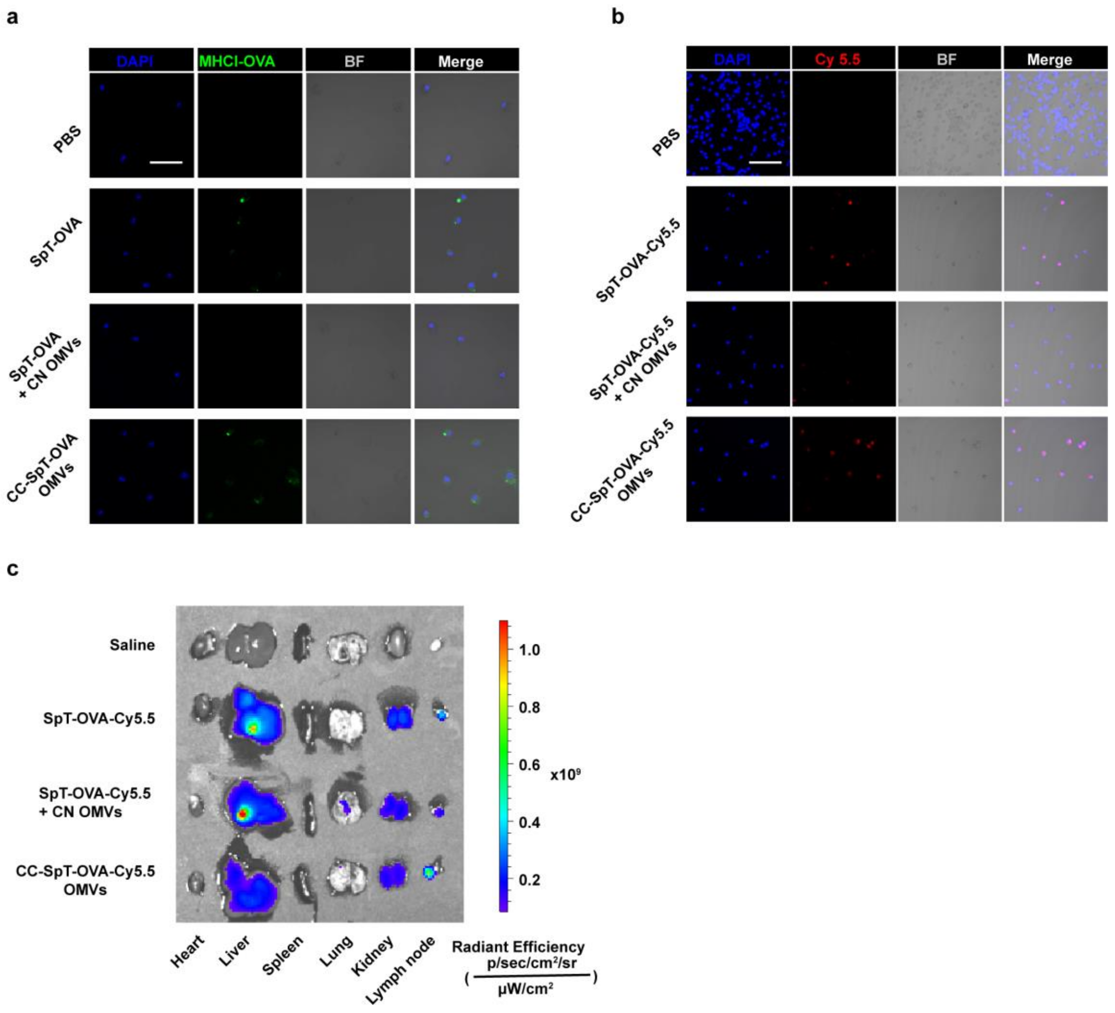

Supplementary Figure 14. Evaluation of lymph node drainage ability of CC OMVs (related to Figure 3f-3h). (a) Confocal microscopy images of antigen presentation by BMDCs incubated with the indicated formulations for 6 h. Cell nuclei were stained blue (DAPI), and MHCI-OVA complexes were stained in green (PE-anti-mouse H-2Kb bound to SIINFEKL). BF, bright field. Scale bar, 50 μm. (b) Uptake of antigens by BMDCs after incubation with the indicated formulations for 6 h as assessed by confocal microscopy. Cell nuclei were stained blue (DAPI), and the antigen was labeled with red Cy5.5. Scale bar, 50 μm. (c) Lymph node accumulation of OMVs *in vivo*. The organs and draining lymph nodes of mice were collected 6 h after intradermal immunization with the indicated formulations to measure the accumulation of Cy5.5 fluorescence.

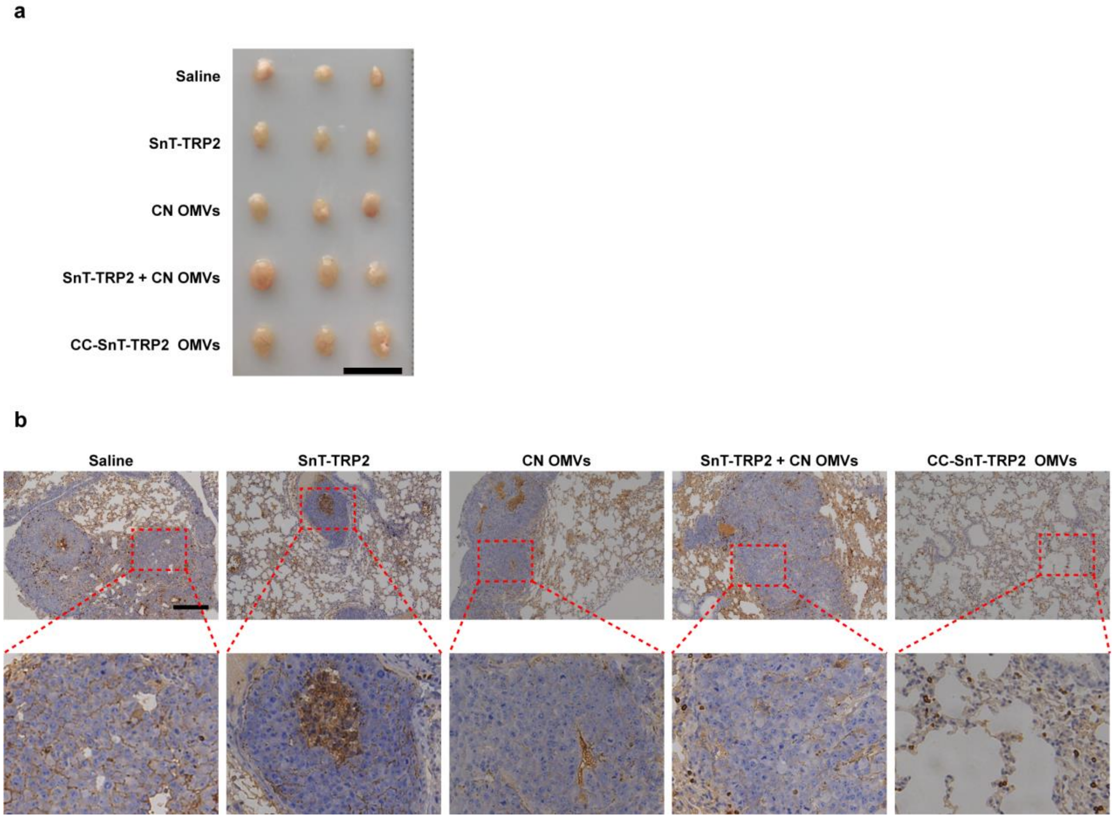

Supplementary Figure 15. Changes in the size of inguinal lymph nodes and CD8^{+} T cell infiltration in the lungs after indicated formulations immunization (related to Figure 4a). (a) The inguinal lymph nodes from mice on day 17 after challenge with B16-F10 melanoma cells and immunization with the indicated formulations were collected and photographed. Scale bar, 1 cm. (b) Immunohistochemical staining of CD8^{+} cells (brown) in the lungs of mice at the end of the treatment period. Scale bar, 200 μm.

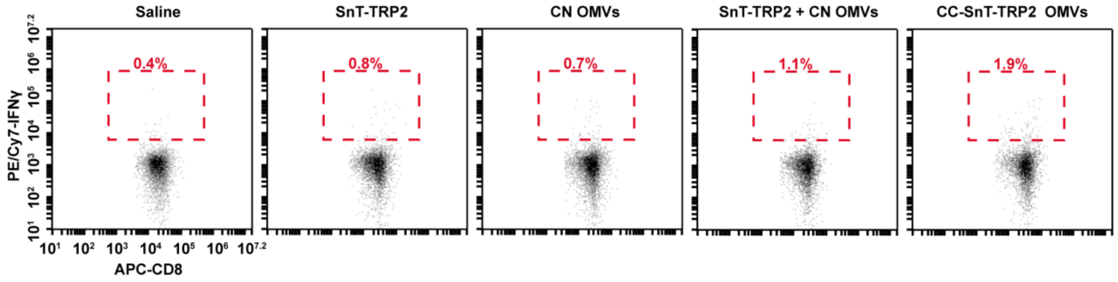

Supplementary Figure 16. CC-SnT-TRP2 OMVs can significantly stimulate antigen-specific response (related to Figure 4f). Representative flow cytometry dot plots of IFNγ^+ cells in $CD3^+CD8^+$ T lymphocytes in splenocytes, re-stimulated with the $TRP2_{180-188}$ antigen, from tumor-bearing mice after vaccination with the indicated formulations.

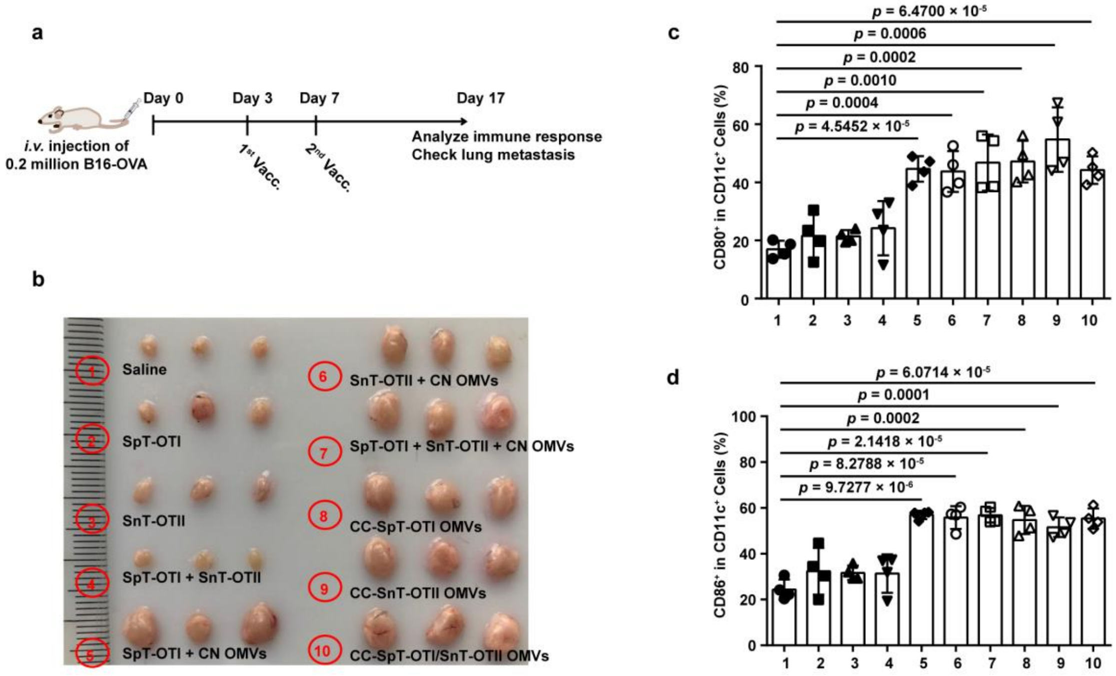

Supplementary Figure 17. Evaluation of DC maturation *in vivo* (related to Figure 5). (a) Schema showing the murine tumor metastasis model utilizing B16-OVA melanoma cells and the timing of vaccination (Vacc.) with the OMVs preparations. C57BL/6 mice were inoculated with B16-OVA melanoma cells (2×10^5 cells/mouse, *i.v.*) and immunized with the indicated formulations on days 3 and 7. Lung metastasis and immune responses were analyzed on day 17. (b) The draining lymph nodes of mice on day 17. The scale is graduated in millimeters. (c) and (d) DC maturation in inguinal lymph nodes on day 17 after tumor cell inoculation and at the end of the immunization period. Flow cytometry was used to determine the percentage of $CD80^+$ (c) or $CD86^+$ (d) cells in the $CD11c^+$ cell subpopulation. All data are presented as mean ± SD (n = 4). Statistical analysis was performed by two-tailed unpaired t-test. Source data are provided as a Source Data file.

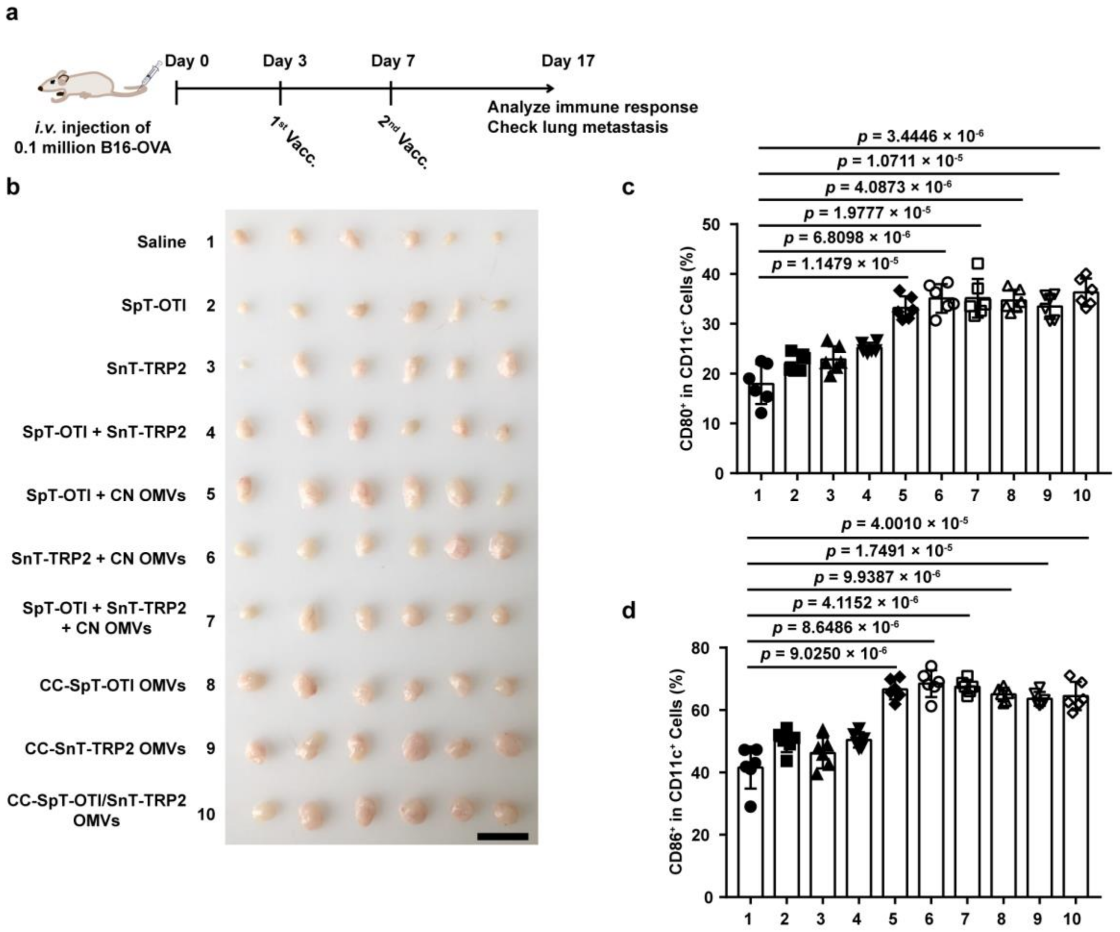

Supplementary Figure 18. Evaluation of DC maturation *in vivo* (related to Figure 6). (a) Schema showing the murine tumor metastasis model utilizing B16-OVA melanoma cells and the timing of vaccination (Vacc.) with the OMVs preparations. C57BL/6 mice were inoculated with B16-OVA melanoma cells (10^5 cells /mouse, *i.v.*) and immunized with the indicated formulations on days 3 and 7. Lung metastasis and immune responses were analyzed on day 17. (b) The draining lymph nodes of mice on day 17. Scale bar, 1 cm. (c) and (d) DC maturation in inguinal lymph nodes on day 17 after tumor cell inoculation and at the end of the immunization period. Flow cytometry was used to determine the percentage of $CD80^+$ (c) or $CD86^+$ (d) cells in the $CD11c^+$ cell subpopulation. All data are presented as mean ± SD (n = 6). Statistical analysis was performed by two-tailed unpaired t-test. Source data are provided as a Source Data file.

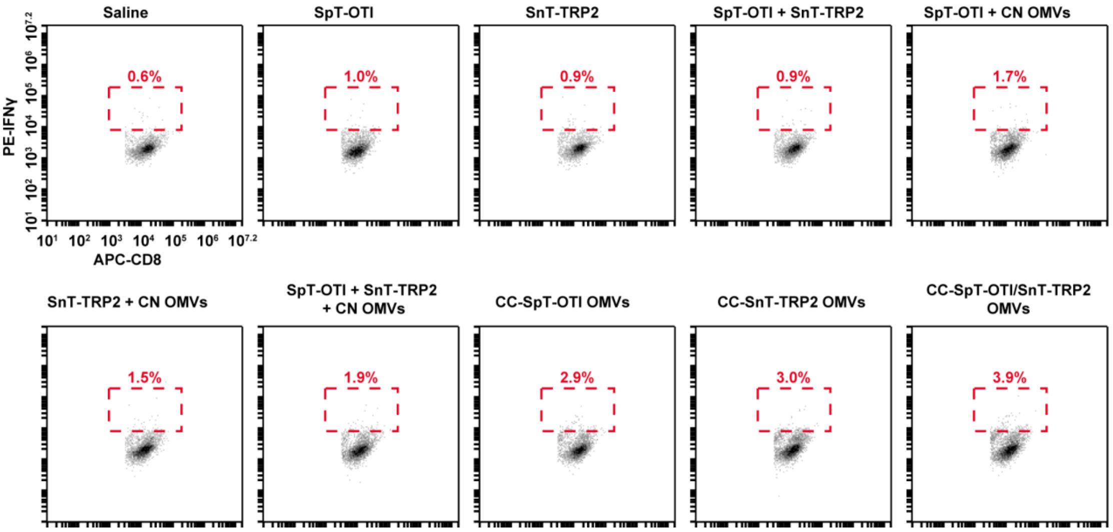

Supplementary Figure 19. Evaluation of antigen-specific immune response induced by CC-SpT-OT1/SnT-TRP2 OMVs (related to Figure 6e). Representative flow cytometry dot plots of IFNγ^{+} cells in CD3^{+}CD8^{+} T lymphocytes in splenocytes, re-stimulated with $OVA_{257-264}$ and $TRP2_{180-188}$ antigens, of tumor-bearing mice after vaccination with the indicated formulations.

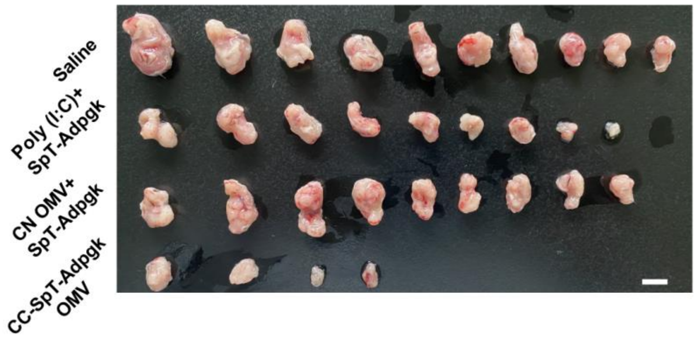

Supplementary Figure 20. Evaluation of anti-tumor immunity in the subcutaneous MC38 tumor model (related to Figure 7a). Tumors were separated from different formulations treated mice on day 29. Scale bar, 1 cm.

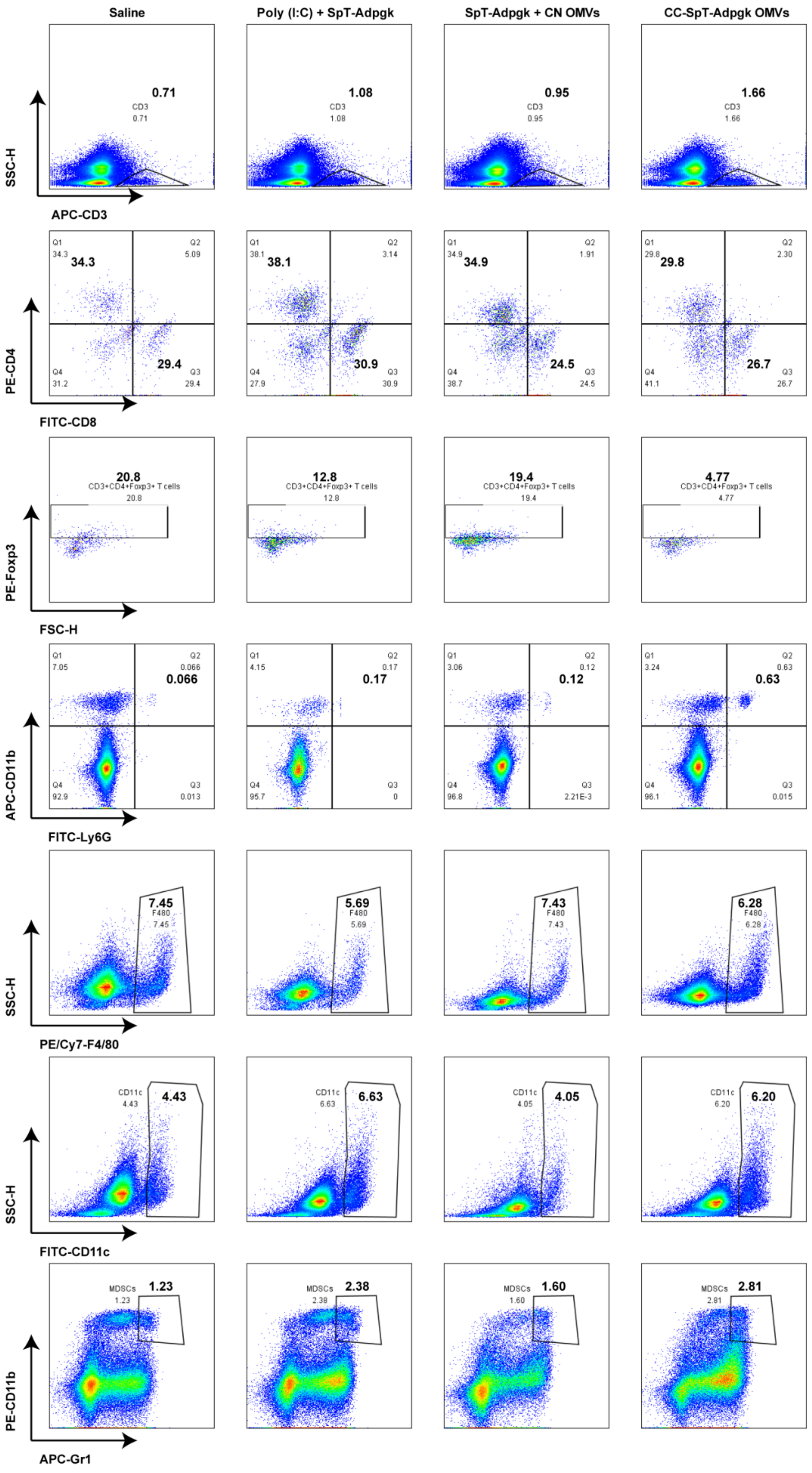

Saline
Poly (I:C) + SpT-Adpgk
SpT-Adpgk + CN OMVs
CC-SpT-Adpgk OMVs
SSC-H
APC-CD3
0.71
1.08
0.95
1.66
PE-CD4
FITC-CD8
34.3
29.4
38.1
30.9
34.9
24.5
29.8
26.7
PE-Foxp3
FSC-H
CD3+CD4+Foxp3+ T cells
20.8
12.8
19.4
4.77
APC-CD11b
FITC-Ly6G
0.066
0.17
0.12
0.63
SSC-H
PE/Cy7-F4/80
F480
7.45
5.69
7.43
6.28
SSC-H
FITC-CD11c
CD11c
4.43
6.63
4.05
6.20
PE-CD11b
APC-Gr1
MDSCs
1.23
2.38
1.60
2.81

Supplementary Figure 21. The infiltration of immune cells in tumor tissues (related to Figure 7e). Representative flow cytometry dot plots of $CD3^+$, $CD3^+CD8^+$, $CD3^+CD4^+$, $CD3^+CD4^+Foxp3^+$ T lymphocytes, activated neutrophils ($CD11b^+Ly6G^+$ cells), macrophages ($F4/80^+$ cells), dendritic cells ($CD11c^+$ cells) and MDSC ($CD11b^+Gr1^+$ cells) in tumor single cell suspensions isolated from the various treatment groups.

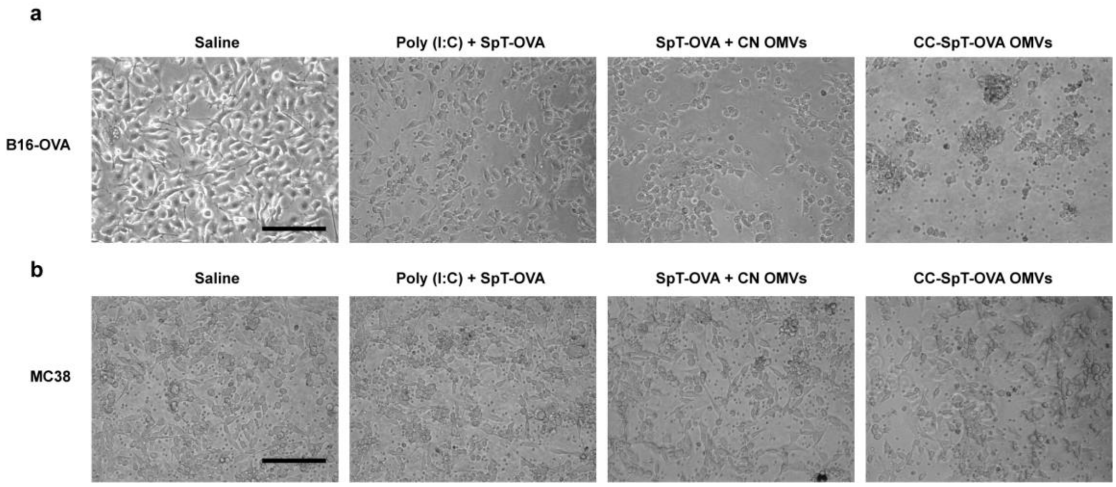

Supplementary Figure 22. Evaluation of antigen-specific killing ability of splencytes from immunized mice (related to Figure 8c-d). The splenocytes from immunized mice were obtained and restimulated with $OVA_{257-264}$. Then, the splenocytes were incubated with B16-OVA cells (a) or MC38 cells (b). Representative images of specific killing are shown, observed by microcopy. Scale bar, 100 μm.

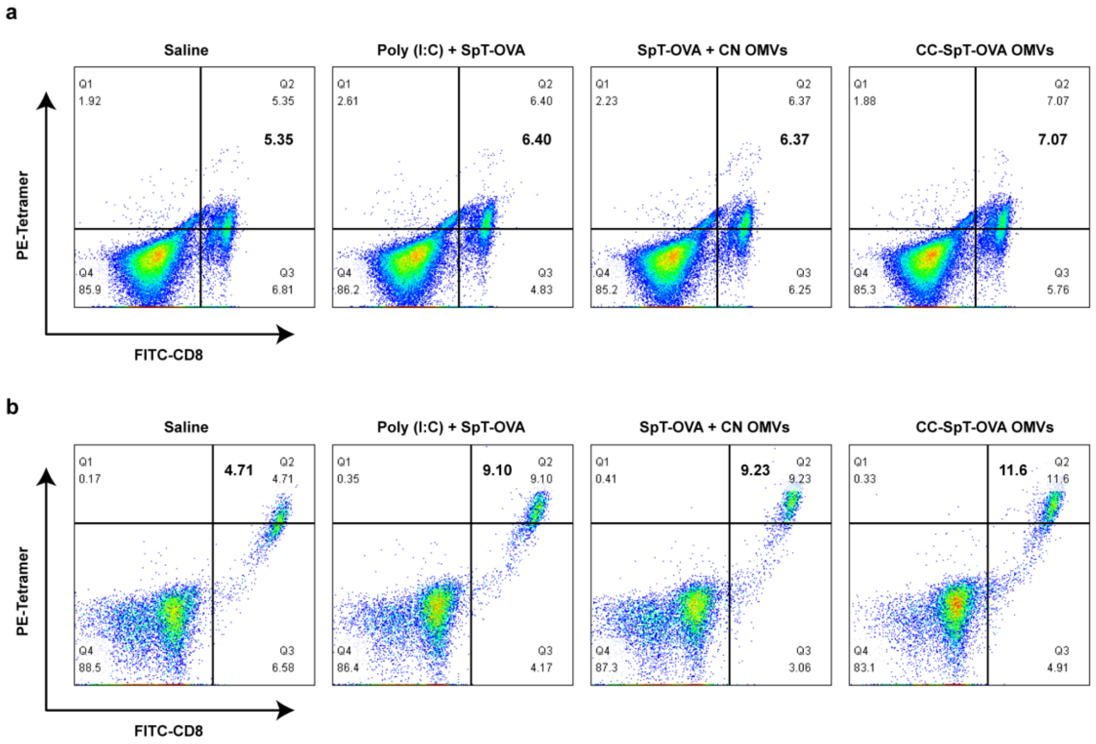

Supplementary Figure 23. Evaluation of antigen-specific immune response (related to Figure 8e-f). Representative flow cytometry dot plots of tetramer$^+$ T cells in splenocytes (a) and blood (b) from immunized mice on days 60.

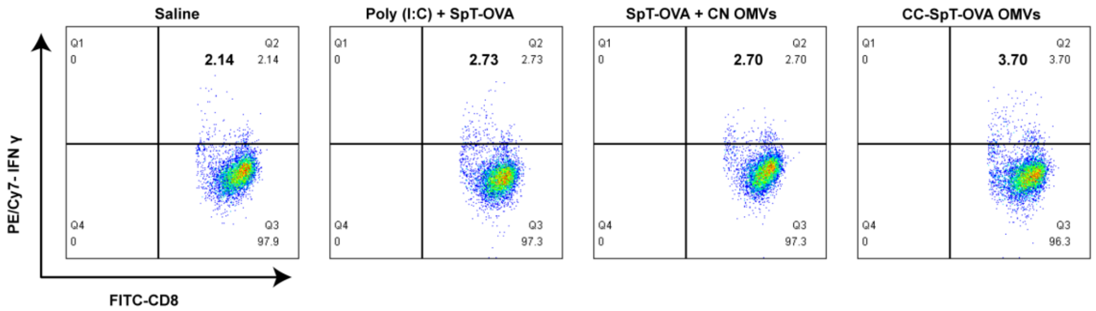

Supplementary Figure 24. Evaluation of antigen-specific immune response (related to Figure 8g). Representative flow cytometry dot plots of IFNγ^{+} cells in CD3^{+}CD8^{+} T lymphocytes in splenocytes re-stimulated with $OVA_{257\text{-}264}$ antigen from immunized mice on days 60.

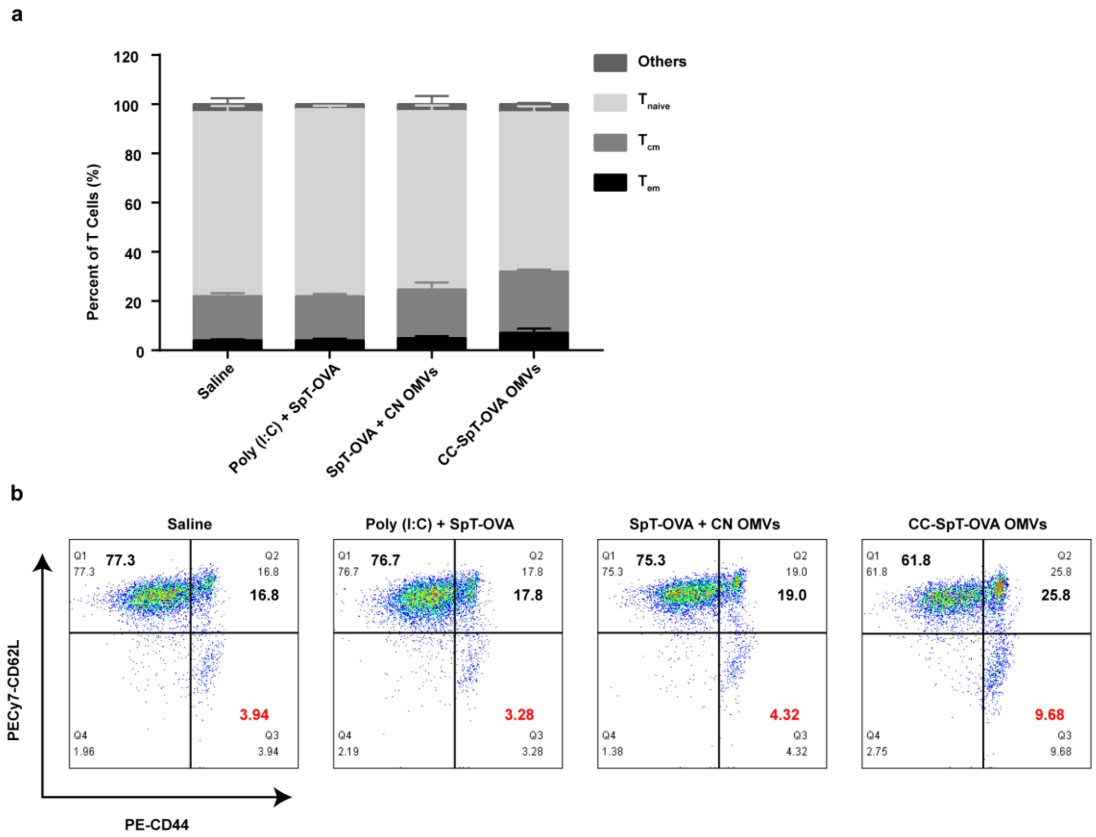

Supplementary Figure 25. Flow cytometry analysis of immune memory (related to Figure 8h). Quantitative analysis (a) and representative flow cytometry dot plots (b) of effector memory T cells (T_{em}, $CD3^+CD8^+CD62L^-CD44^+$), central memory T cells (T_{cm}, $CD3^+CD8^+CD62L^+CD44^+$) and *naive* T cells (T_{naive}, $CD3^+CD8^+CD62L^+CD44^-$) in splenocytes on days 60, showing CC-SpT-OVA OMVs-induced T cell memory. All data are presented as mean ± SD (n = 5). Source data are provided as a Source Data file.

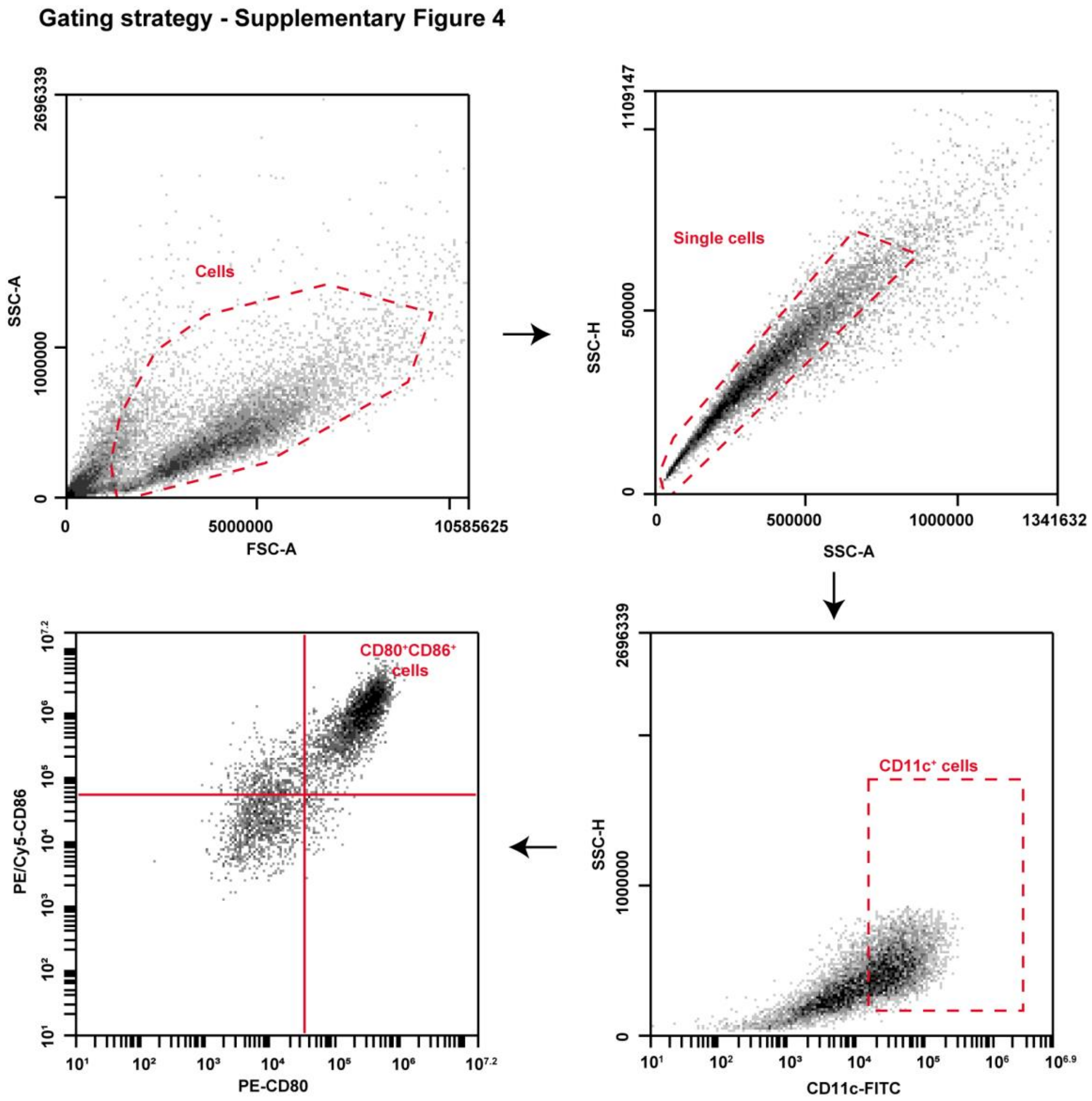

Supplementary Figure 26. Gating strategy for Supplementary Figure 4.

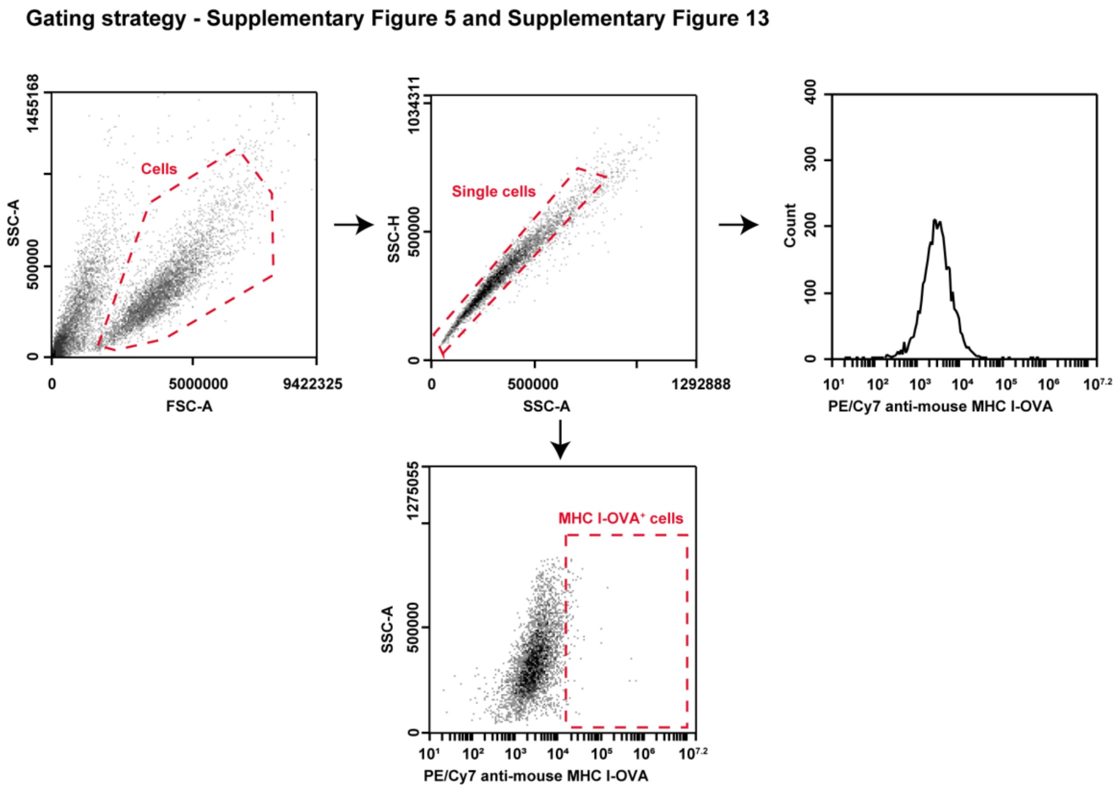

Supplementary Figure 27. Gating strategy for Supplementary Figure 5 and Supplementary Figure 13.

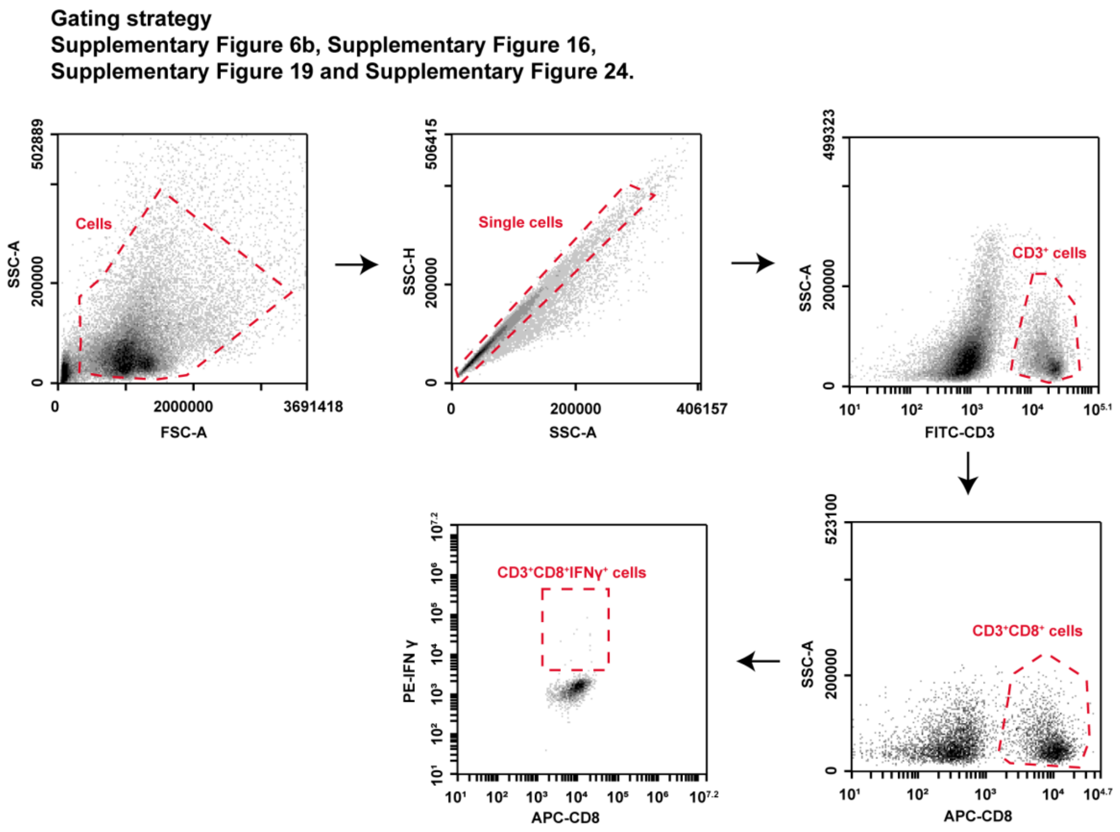

Supplementary Figure 28. Gating strategy for Supplementary Figure 6b, Supplementary Figure 16, Supplementary Figure 19 and Supplementary Figure 24.

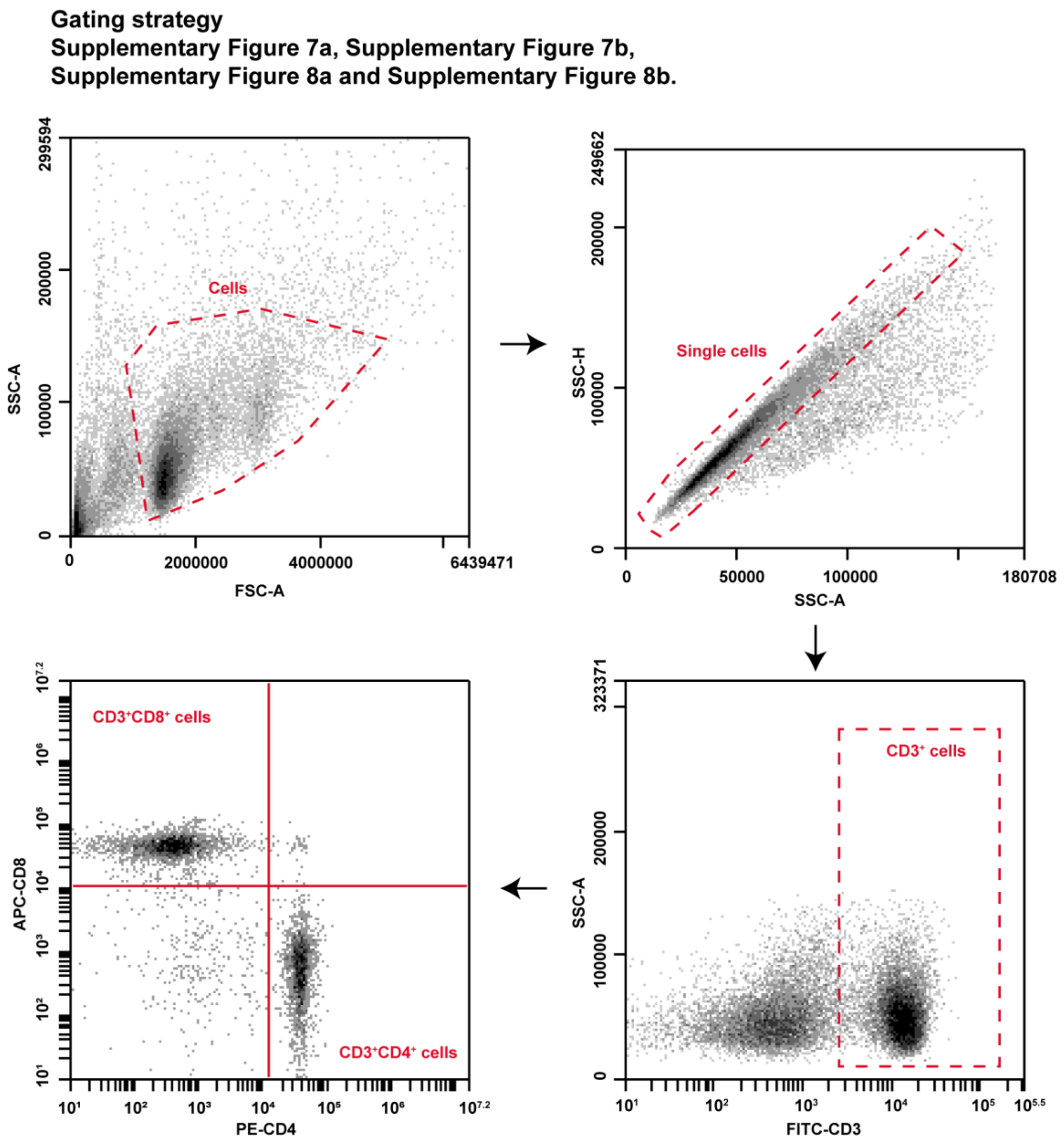

Supplementary Figure 29. Gating strategy for Supplementary Figure 7a, Supplementary Figure 7b, Supplementary Figure 8a and Supplementary Figure 8b.

Gating strategy-Supplementary Figure 21

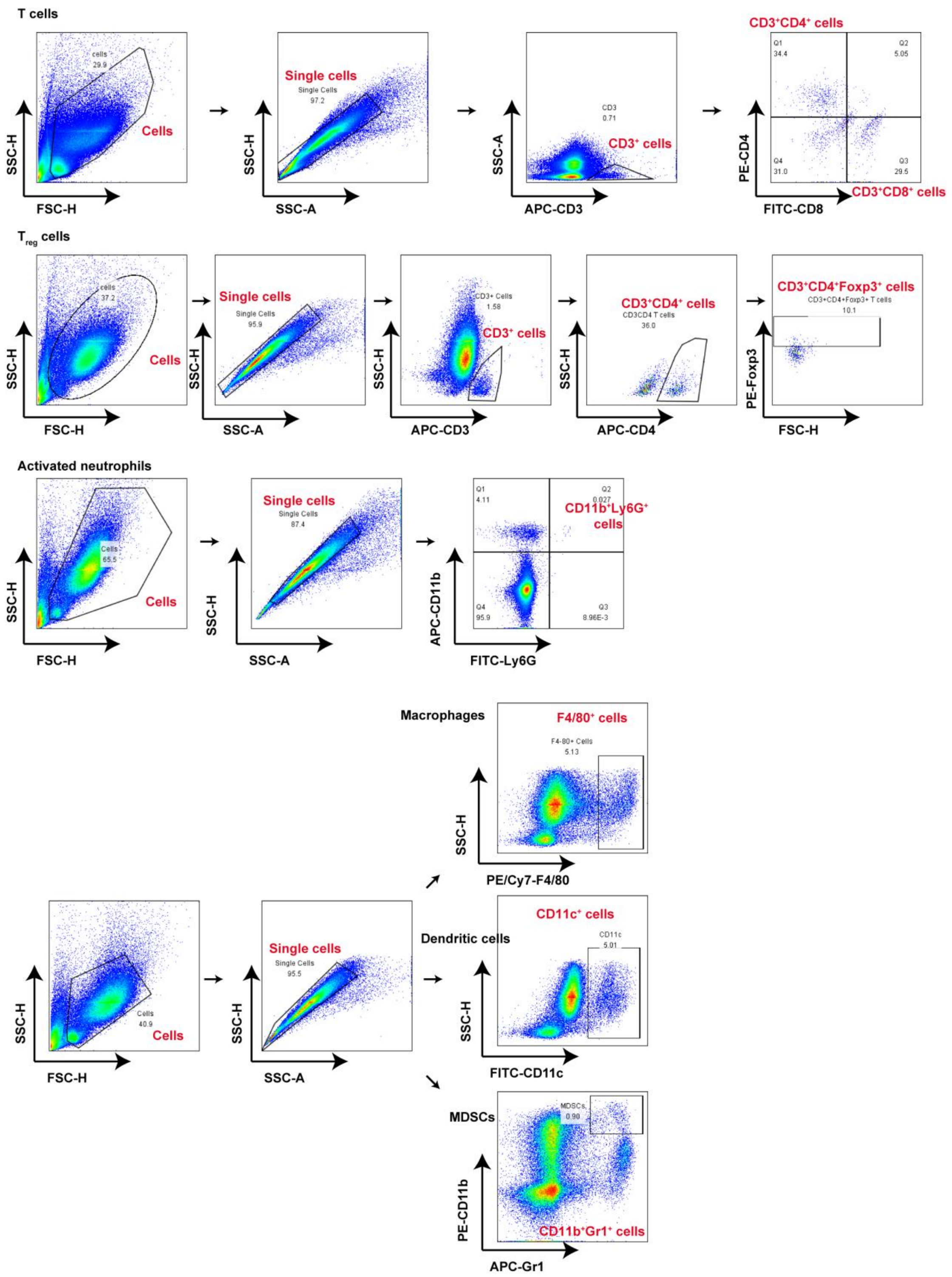

Supplementary Figure 30. Gating strategy for Supplementary Figure 21.

Gating strategy-Supplementary Figure 23a and Supplementary Figure 23b

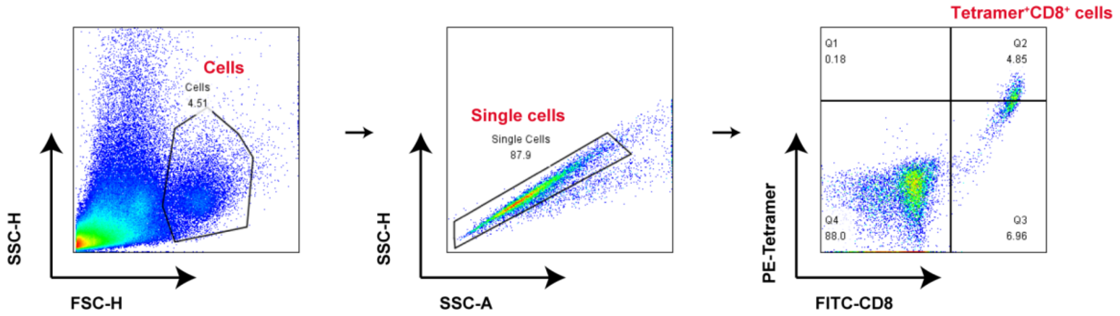

Supplementary Figure 31. Gating strategy for Supplementary Figure 23a and Supplementary Figure 23b.

Gating strategy-Supplementary Figure 25b

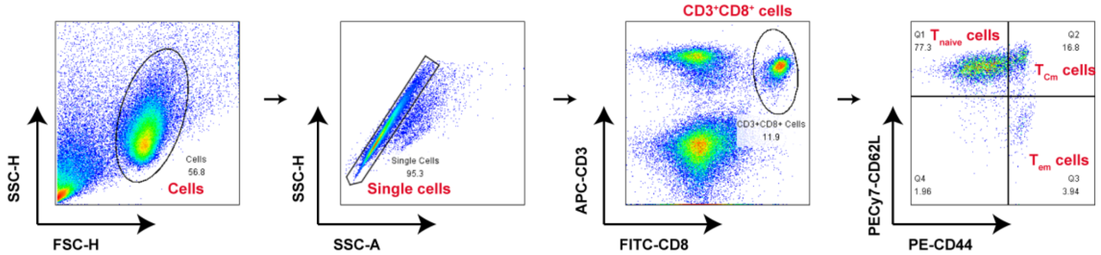

Supplementary Figure 32. Gating strategy for Supplementary Figure 25b.
